# Acknowledgment to Reviewers of *Diagnostics* in 2020

**DOI:** 10.3390/diagnostics11020148

**Published:** 2021-01-20

**Authors:** Diagnostics Editorial Office

**Affiliations:** MDPI AG, St. Alban-Anlage 66, 4052 Basel, Switzerland

Peer review is the driving force of journal development, and reviewers are gatekeepers who ensure that *Diagnostics* maintains its standards for the high quality of its published papers. Thanks to the cooperation of our reviewers, in 2020, the median time to first decision was 16 days and the median time to publication was 33 days. The editors would like to express their sincere gratitude to the following reviewers for their precious time and dedication, regardless of whether the papers were finally published:
Aarntzen, ErikLopez-Rincon, AlejandroAbdelbasset, Walid KamalLópez-Urrutia, EduardoAbdul-Rahman, Omar A.LoPresti, PatriziaAbe, TomoyukiLops, DiegoAbomaray, Fawaz MohamedLorenz Risch, LorenzAcampa, MaurizioLorusso, AlessioAcharjee, AnimeshLosi, Maria AngelaAchiron, AsafLu, Chia-FengAcién, PedroLu, JingAcker, Jason P.Lu, LingengAcri, GiuseppeLu, ShuiyuAdam, Margaret P.Luangmonkong, TheerutAdamczewski, ZbigniewLubas, ArkadiuszAdamiok-Ostrowska, AnnaLubecka-Pietruszewska, KatarzynaAdamopoulos, PanagiotisLucarelli, GiuseppeAfat, SaifLucchese, AlessandraAfrantou, TheodoraLudmir, Ethan B.Agarwal, SumitLukasiewicz, AleksanderAggeli, ConstantinaLukoševičius, ArūnasAgresta, FerdinandoLunt, PeterAhmad, KhurshidLuo, Ji-DungAhn, YongLuo, XianAi, YongLuzzago, StefanoAich, SatyabrataM’kacher, RadhiaAizenberg, MicheleMa, Suk LingAkagi, SatoshiMaas, MarioAlaimo, AlessandroMaboni, GrazieliAl-antari, Mugahed A.Maccio, AntonioAlbano, DomenicoMacek, PawelAlberti, DanieleMachairiotis, NikolaosAlbertolle, Matthew E.Macq, BenoitAlbu, SilviuMadani, AfarineAlcalde, MireiaMadea, BurkhardAlcántara-Ortigoza, Miguel AngelMadeddu, PaoloAlexander, AdrianMadonov, PavelAli, Lamiaa M. A.Madrigal, IreneAli, ZulfiqurMaeda, KazuhiroAl-Imam, AhmedMaffei, AngeloAlizargar, JavadMagan-Fernandez, AntonioAljabali, Alaa A. A.Magi-Galluzzi, CristinaAlkan, MichaelMagnaldo, ThierryAlkatout, IbrahimMahan, J. RobertAlkhateeb, AbedalrhmanMahshid, SaraAllen, Richard C.Maiega, HamidouAl-Maawi, SarahMair, JohannesAlmansa, InmaculadaMais, ValerioAlmeida, LuisMajchrzak, TomaszAloisi, Anna MariaMajewski, SebastianAlongi, PierpaoloMajolino, DomenicoAlpar, DonatMajtner, TomášAlpar, OrcanMajumder, MrinmoyeeAltamura, ClaudiaMakavos, GeorgeAltini, CorinnaMakieva, SofiaAltintas, Mehmet M.Makki, MalekAltmann, FriedrichMakkiya, MohammedÁlvaro, TomásMaksemous, NevenAlzubaidi, LaithMalafoglia, ValentinaAmadio, PeterMalagelada, FrancescAmado, SandraMalaguarnera, MicheleAmaricai, ElenaMalapelle, UmbertoAmedei, AmedeoMalatino, LorenzoAmin, Nivee P.Malek, LukaszAmit, MoranMalla, SpundanaAmorim, JoãoMalli, FoteiniAmornyotin, SomchaiMallidi, SrivalleeshaAmzar, DanielaMallineni, Sreekanth KumarAna Maria Cristina, TancuMalouf, CamilleAnagnostis, AthanasiosManali, EffrosyniAnania, Maria ChiaraMancusi, CostantinoAnanthakrishnan, Soundaram JeevarathinamMancuso, SalvatriceAndaloro, ClaudioMandal, SubhamoyAndica, ChristinaMandato, ClaudiaAndrade De Jesus, DaniloManenti, AntonioAndrearczyk, VincentManfredini, FabioAndreetti, ClaudioMangino, GiorgioAndrius, KazlauskasMango, LucioAndrukhov, OlehMangogna, AlessandroAndrzej, NowickiManini, PaolaAnemona, LuciaMannelli, LorenzoAng, Eng TatMännistö, TuijaAngelini, CorradoMansourian, MarjanAngelousi, AnnaMäntele, WernerAngheben, AndreaMantripragada, Venkata P.Anghel, LarisaMarateb, Hamid RezaAnitescu, CosminMarc, LaineAnnaswamy, Thiru M.Marchi, AntonellaAnsani, LuciaMarcon, MagdaAntignani, Pier LuigiMarcu, LoredanaAntigua, Khristine Joy C.Mare, RuxandraAntonakakis, MariosMarella, Gian LucaAntonela Antoniu, SabinaMargalit, IliAntoniu, Sabina AntonelaMaria, Naomi I.Antony, Benny S. EathakkattuMariano, CaratozzoloAntony, ReubenMarinelli, LucaAnwar, Syed MuhammadMarino, BrunoAoki, MitsuhiroMario, PagnoniAoki, TakatoshiMarkić, JoškoAoude, LaurenMarkiewicz, AleksandraApplegate, TanyaMarkman, MaurieApra, CarolineMarkopoulos, AnastasiosAran, VeronicaMarkopoulos, Georgios S.Arandjelović, OgnjenMarkou, AthinaArason, AdalgeirMarks, Robert S.Ardeleanu, ValeriuMármol, InesArena, AlessandroMarotte, HubertArgula, Rahul GuptaMarquering, Henk A.Arizono, ShigekiMarques, João GamaArmellini, EliaMarques, TaniaArmengot-Carceller, MiguelMarrelli, MassimoArnedo, MariaMarshall, Andrew G.Arosio, PaoloMartaindale, SarahArtigas, CarlosMarteau, Philippe R.Arul, MichaelMartin, Diana L.Ashida, ReikoMartin, MariaAsimaki, AngelikiMartinelli, FabioAsmundo, AlessioMartinez-Camblor, PabloAso, YoshimasaMartini, KatharinaAssi, MohamadMartins, Andre F.Assinder, StephenMarto, JoanaAta-Ali, JavierMartucci, GennaroAuriti, CinziaMarzano, FlavianaAushev, VasilyMasahiro, SatoAvanzo, MicheleMasci, LorenzoÁvila, JulioMascitti, MarcoAyaki, MasahikoMaspero, CinziaAyat, NadiaMastoraki, AikateriniAydede, Sema K.Mastropietro, AlfonsoAyoub, AshrafMasumoto, HidetoshiAzizian, AzadehMasuzaki, RyotaBabar, GhufranMatczuk, Anna KarolinaBacalbaşa, NicolaeMaterazzi, StefanoBaffy, GyorgyMateus, Maria L.Bagci, UlasMatheve, ThomasBagiu, Iulia-CristinaMathew, Ashish J.Bahmad, HishamMathiessen, AlexanderBaig, ImranMatozaki, TakashiBailey, SimonMatsubara, Edson TakashiBainbridge, JacciMatsuda, AtsushiBajan, SarahMatsumoto, NaokiBajwa, AmandeepMatsuoka, TeruyukiBakhshinejad, AliMatsusaki, TakashiBakht, MartinMatsushima, TakashiBalaban, Daniel VasileMatsuzaki, KentaroBaldacci, FilippoMattapallil, JosephBaloyannis, Stavros J.Matynia, AnnaBaltzer, Pascal A. T.Maurea, NicolaBamatraf, Saeed M.Maurea, SimoneBandres-Ciga, SaraMaw, Anna M.Baniahmad, AriaMayer, PhilippBansal, AdityaMayer, RupertBär, SéverineMazza, ElisaBaratella, ElisaMbagwu, Smart IkechukwuBarauskas, GiedriusMcClure, PatrickBarbee, Lindley A.McDermott, John H.Barberis, MassimoMcKay, TinaBarbosa, Ana IsabelMcmorrow, TaraBarbosa, Flávia LeãoMcNeill, David MorenoBarca, AmilcareMcsharry, CharlesBarda, NoamMeder, RogerBarnes, AnnaMedipally, DineshBarnett, BradMędrzycka, WiolettaBarra, FabioMeduri, AlessandroBarran, Perdita E.Medvedev, Ilya NikolaevichBar-Shalom, RachelMeehan, KatieBartel, SabineMehariya, SanjeetBartnik, EwaMehta, NoshirBartoli, FrancescoMehta, SunaliBarton, Matthew J.Mehterov, NikolayBartulos, OscarMei, ShenglinBarua, AnimeshMeignan, MichelBasak, SujitMeimandi, Kiana JafariBassareo, Pier PaoloMelaiu, OmbrettaBatchuluun, GanbayarMelas, MarilenaBattistelli, CeciliaMeleady, PaulaBauckneht, MatteoMelling, NathanielBaumrucker, StevenMelo, Bernardete F.Baxter, EvaMenahem, SolomonBay, PeterMercadal, LucileBeasley, ShannonMerkely, BélaBechsgaard, ThorMerks, HansBechter, KarlMermis, Joel D.Bednarz-Knoll, NataliaMerolla, FrancescoBeer, Ambros J.Merrick, DanielBehringer, ErikMesa, FranciscoBel’skaya, LyudmilaMesalam, AhmedBelcher, John R.Meshram, NirvedhBellin, Marie FranceMessineo, LudovicoBelluzzi, ElisaMestrovic, TomislavBender, Steven D.Metaxas, GeorgeBenko, ZsofiaMetovic, JasnaBennardo, FrancescoMetzinger, LaurentBennardo, LuigiMeulstee, Jene W.Beňuš, RadoslavMeyer, Hans-JonasBenyhe, SandorMia, SobujBenzaquen, MichaelMiazek, ArkadiuszBerardo, ClarissaMichael, MichaelBeresford, DavidMichálek, PavelBerezin, AlexanderMigita, OhsukeBergandi, LoredanaMihaicuta, StefanBergougnoux, AnneMikaïl, NidaaBernabéu, CarmeloMiki, KojiroBerretti, StefanoMiki, YasuhiroBertelli, ElenaMikropoulos, ChristosBerthelot, LaurelineMileti, IlariaBertoli, GloriaMiller, Sara E.Bertomeu-González, VicenteMiller, Tamara P.Bertucci, EmmaMinaga, KosukeBevc, SebastjanMincher, DavidBhat, Ambarish P.Minet, OlafBhatia, VikramMiotla, PawelBhute, ShrikantMirzakhani, HoomanBiałynicki-Birula, RafałMiserocchi, GiuseppeBianconi, FrancescoMishra, Jay S.Bikov, AndrasMisiak, BłazejBinda, CeciliaMisiolek, MaciejBiswas, ArijitMitchell, John S.Biswas, SantanuMitchell, Ulrike H.Bis-Wencel, HannaMitrofanova, AllaBlanc, DominiqueMitrut, RaduBlank, Andreas ChristianMiyata, YasuyoshiBlanton, Robert M.Miyazaki, MasaruBlasi, ElisabettaMiyoshi, NorioBłaszczyszyn, MonikaMizrahi, JosephBlaumeiser, BettinaMlynarska, AgnieszkaBleilevens, ChristianMo, Lein-RayBloch, Karin MarkenrothMo, MakotoBoaventura, PaulaMoadel, Renee M.Boccolini, DanielaMoaven, OmeedBödör, CsabaMoccia, MarcelloBöer, KlasModahl, CassandraBohannon, Richard W.Mohamed, Salah A.Bolboacă, Sorana D.Mohsin, SadiaBoldorini, RenzoMolina-Alarcón, MilagrosBologna, RonellMolina-Leyva, AlejandroBonda, TomaszMoller, IngridBonetto, AndreaMøller, SørenBongiorno, DafneMoncayo, RoyBonham, JimMonda, EmanueleBonifazi, MartinaMonsalve, MariaBordonaro, MichaelMonsuez, Jean-JacquesBorghesi, AndreaMontagne, AxelBorgia, Jeffrey A.Monteiro, LuisBorisova, EkaterinaMonteiro, SílviaBoros, LaszloMoore, Matthew D.Borovac, Josip A.Moore, Ronald B.Borroni, DavideMoore, StuartBosch, XavierMorales Narváez, EdenBose, TanimaMoran, Cesar A.Botella, Luisa MaríaMoran, OscarBotnar, ReneMoretti, MatteoBotti, GerardoMorga, RafałBotton, ThomasMori, KiyoshiBoubchir, LarbiMorio, YoshiteruBouhrara, MustaphaMornos, CristianBowling, Heather L.Moro, AlessandroBoysen, ReinhardMorris, DanielBozso, Sabin J.Morris, UlrikaBracht, ThiloMoscalu, MihaelaBrady, AdrianMotakis, EfthymiosBrady, ZoeMounien, LourdesBragazzi, Nicola LuigiMozos, IoanaBraicu, CorneliaMrówczyńska, MariaBrak, IvanMucci, VivianaBratashov, Daniil N.Mueller, GeoffreyBrcic, LukaMuhammad, KhanBrcic, MarioMukaida, NaofumiBressem, Keno-KyrillMüllauer, LeonhardBreuker, AnjaMumoli, NicolaBrieger, AngelaMuniraj, NethajiBrigante, GiuliaMuniyan, SakthivelBroderick, Patricia A.Muñoz-Berbel, XavierBroggi, MorganMunteanu, Florentina-DanielaBrogi, SimoneMurakami, NaokaBrogna, ClaudiaMurata, SoichiroBromberg, Mark B.Muro, ShigeoBrosens, ErwinMurph, MandiBrown, Christopher LyonMurphy, Caroline S.Broza, YoavMurphy, RonanBrozek-Pluska, BeataMuscatiello, NicolaBrun, EmmanuelMußbacher, MarionBruneel, ArnaudMuthupillai, RajaBrunetti, OronzoNabeta, TakeruBruno, Antonio GiulioNachmias, BoazBrustmann, HermannNaeije, RobertBruzzese, DarioNagai, NorihiroBryere, JoséphineNagaoka, MasanoriBuckley, LizNagaraja, Haikady N.Bucolo, ClaudioNagarajan, PrabakaranBudzevich, Mikalai M.Nagayasu, EijiBudzik, Jean-FrançoisNagy, MagdolnaBueno-Campaña, MercedesNaicker, NishaBueno-Crespo, AndrésNakachi, TatsuyaBuffi, Nicolò MariaNakagami, GojiroBujan, JuliaNakagawa, NaokiBüki, AndrásNakahara, IchiroBultot, LaurentNakai, YousukeBundschuh, Ralph A.Nakajima, TakahitoBungau, Simona GabrielaNakamura, YoshiyukiBuonaguro, Elisabetta FilomenaNakanishi, HiroyukiBurattini, LauraNakano, ToshiakiBurger, Michael C.Nakayama, HaruoButkauskas, DaliusNakayama, HideakiBuza, KrisztianNakayama, JunByeon, HaewonNambiar, Dhanya K.Byoung-Hee, LeeNanetti, LorenzoCaggiati, AlbertoNaňka, OndřejCaiati, CarloNaotake, TsudaCaivano, DomenicoNapoletano, ChiaraCalcagni, GiulioNapoli, PietroCaldara, MarinaNarayan, PrakashCaldarella, CarmeloNardella, ElizabethCaliò, AnnaNardini, RobertoCallegaro, AnnapaolaNascimento, Gustavo G.Caltabiano, RosarioNasierowska-Guttmejer, AnnaCalvano, Cosima DamianaNatarajan, Sathish KumarCalvieri, StefanoNath, Narayan Chandra DebCalvo Lobo, CésarNatsis, KonstantinosCamara Serrano, JuanNault, Marie-LyneCameselle-Teijeiro, Josè ManuelNavakauskienė, RūtaCampagna, GiuseppeNavarro, EstanisCampana, Luca GiovanniNavran, ArashCampus, GuglielmoNayak, NiharCandela, Diaz-CanestroNaydenov, StefanCândido Carvalho, KátiaNazzaro, GianlucaCannella, RobertoNeginskaya, Maria A.Canti, ValentinaNemeș, Roxana MariaCanty, David J.Németh, BalázsCao, JianNemeth, NorbertCaparros-Gonzalez, Rafael A.Nenna, AntonioCapoccia, MassimoNeogi, UjjwalCaponio, Vito Carlo AlbertoNeri, LucaCaporali, LeonardoNeri, ManuelaCappelletti, VeraNesbitt, Warwick S.Captur, GabriellaNestrasil, IgorCapurso, GabrieleNeubauer, HansCarbone, AntoninoNeuhaus, Ain A.Carbone, MicheleNevler, NaomiCarboni, NicolaNezhat, Farr RezaCarinelli, SilvestroNguyen, AnhCarney, A SimonNguyen, BinhCaroli, AnnaNguyen, TinCarpenter, LewisNguyen, TrieuCarpi, SaraNi, YichengCarraro, UgoNiaura, GediminasCarrier, PaulNicolai, EleonoraCarriero, AlessandroNiczyporyk, Jowita SamantaCarrilho, EuniceNie, LimingCarrizzo, AlbinoNiedzialkowski, PawelCarugno, JoseNii, MasafumiCaruntu, ConstantinNikaki, AlexandraCaruso, DamianoNikita, EfthymiaCarvalho, BeatrizNikolai, Bryan C.Casals, GregoriNikolaidis, AthanasiosCasari, AliceNikolaidis, Pantelis T.Casas, FrançoisNikolic, DejanCasas, Rosa M.Nikolova, IrinaCascini, Giuseppe LucioNilghaz, AzadehCasella, MichelaNilsson, Inga-LenaCaserta, DonatellaNilvebrant, JohanCasiraghi, ElenaNioi, MatteoCastresana, Javier S.Niraula, Boris B.Castro, AndreNishida, NaokiCastro, PedroNishii, TatsuyaCatalano, AntoninoNishio, MizuhoCatanzaro, John N.Nishioka, ShintaCaterino, UmbertoNiu, TianhuaCavalli, LoredanaNivedita, NiveditaCaviglia, Gian PaoloNixon, Daniel W.Cavoretto, PaoloNociari, MarceloCazzolla, Angela PiaNogami, MakotoCebova, MartinaNohe, AnjaCécile, DelettreNonnemacher, Michael R.Celi, AlessandroNoorani, ImranCellina, MichaelaNopp, AnnaCeppi, LorenzoNorcic, GregorCerrolaza, Juan J.Nordman, InaCervantes, JorgeNoríaki, TomuraCervigón, RaquelNosch, Daniela SonjaChace, Donald H.Noszczyk, Bartłomiej HenrykChadwick, PhilipNoventa, MarcoChadwick, Raymond J.Novo-Veleiro, IgnacioChan, KannieNovroski, NicoleChan, Kwok-HungNovy, JanChang, Chia-ChenNowicki, MichałChang, HyeyoonNucera, EleonoraChang, Jia-FengNucera, RiccardoChang, Ke-VinNucifora, GaetanoChang, Shin-TsuNurwidya, FarizChang, Yao-LungNuvoli, SusannaChao, Chia-TerNwabudike, Lawrence ChukwudiChao, Chien-MingNylander, KarinChao, Day-YuNyman, JeffryCharabidze, DamienO’Loughlin, DeclanChaudhary, PankajOakey, ZackeryChauhan, NeerajObermüller, NicholasChaveeva, PetyaOchoa-Reparaz, JavierChavez-Santoscoy, Rocio AlejandraOh, Hoon KyuChen, ArgonOh, JungHwanChen, Chien-ChinOhgami, Robert S.Chen, Chun-JungOhko, KentaroChen, Hsiang-YinOhlemiller, Kevin K.Chen, HuarongÖhrfelt, AnnikaChen, Jyh-ChengOkada, HiroshiChen, Ke-ChengOkajima, HideakiChen, Kevin T.Okonogi, NoriyukiChen, Kuan-FuOldan, JorgeChen, Kuan-HsingOleksiewicz, UrszulaChen, LibenOliva, FrancescoChen, MingshengOliveira, Arlindo L.Chen, Shing-JyeOliveira, Paula A.Chen, Tai-HengOliver, EduardoChen, XiaoyanOlivera, MarcoChen, Yann-JangOlivetti, Elena CarlottaChen, Yei-TsungOlmedillas-López, SusanaChen, Yu-ChunOltra, ElisaChen, ZhitongOmar, Syed HarisCheng, Chun-AnOmoumi, PatrickCheng, Juei-TangOmura, JunichiChepelev, LeonidOno, YukoCheungpasitporn, WisitOriol, AlbertChi, GeraldOrlacchio, ArturoChi, Yung-WeiOrosz, TamásChiaramello, AnneOrtega, Miguel AngelChiaramonte, RitaOrtiz, CarmenChicklore, SugamaOrzechowski, ArkadiuszChien, Yin-HsiuOshima, YusukeChigbu, De Gaulle I.Osna, NataliaChinegwundoh, FrancisOsztheimer, IstvánChing, Congo Tak ShingOthman, Ahmed E.Chintalapudi, SumanaOttaiano, AlessandroChioncel, OvidiuOttenheijm, Ramon P. G.Chitale, ShalakaOuahabi, AbdeldjalilChizzolini, CarloOvsepian, Saak V.Chlanda, PetrOwczarczyk-Saczonek, AgnieszkaCho, JaehoPaal, PeterCho, Kang SuPaço, MariaChoi, HojongPafili, KalliopiChoi, Joon HyukPagani, MarcoChoi, Woo JunePahk, KisooChong, Cher-RinPal, DebjaniChong, YosepPalazón-Bru, AntonioChoo, Pei LingPalencia-Madrid, LeireChoudhary, SanjeevPalma, MarziaChowdhury, Imran HussainPalmucci, StefanoChrcanovic, BrunoPalumbo, CarlottaChristopoulos, PetrosPalumbo, GiuseppeChroni, ElisabethPampush, JamesChu, Chun HungPan, LuyingChuah, Seng-KeePanchapakesan, BalajiChuang, Wen-YuPanda, SantoshChulanov, VladmirPandit, HarshulChun, Dong-ilPanzuto, FrancescoChung, Chi-LiPaone, GaetanoChung, Yong-AnPapageorgiou, ElpinikiCianci, PasqualePapageorgiou, Ismini E.Ciancio, GaetanoPapalia, RoccoCianciolo, GiuseppePapamichail, KonstantinosCicone, FrancescoPapanas, NikolaosCiebiera, MichalPapandrianos, NikolaosCilia, GiovanniPapathanakos, GeorgiosCillero-Pastor, BertaPapathanasiou, MariaCiobica, AlinPaplińska-Goryca, MagdalenaCipakova, IngridPapp, JohnCipolloni, LuigiParenti, IlariaCivit, AntonParikesit, Arli AdityaClarke, Luka AlexanderParikh, HemangClemens, Robert K.Parimon, TanyalakClement, YuenParis, DeboraCoccheri, SergioPark, HoonCocco, StefaniaPark, HyesunCofta, SzczepanPark, JeongjinCohen, Michael B.Park, Kay-HyunColetta, RicardoPark, MinColicchia, MichelePark, So HyunCollado, AidaParlatescu, IoaninaCollier, AndrewParnetti, LucillaCollini, FedericaParravano, MariacristinaColomo, LluísPasta, GianluigiColon Saez, JosePasto, AnnaColosi, Horațiu AlexandruPastores, Gregory M.Colosimo, CarloPathak, DhrubaConceicao, RaquelPatowary, AshokConti, PioPavesio, CarlosConti, ValeriaPavlidis, EfstathiosContreras-Aguilar, María DoloresPavlik, Edward J.Coppel, YannickPavlov, Chavdar S.Coraci, DanielePavlova, ElenaCorda, AndreaPavlu, DagmarCornejo, AlbertoPawar, Shrikant D.Cornelis, FrançoisPawlak, KatarzynaCornelius, DenisePayabvash, Seyedmehdi Mehdi D.Corrigan, Damion K.Payne, Karl Frederick BraekkanCorris, Paul A.Peckham, Gabriel D.Cosby, LouisePecorelli, AnnaCosmin Mihai, VesaPei, YonggangCosta, JoanaPeitsidis, PanagiotisCosta, PedroPellacani, GiovanniCottereau, Anne-SégolènePellegrini, MarcoCoucke, WimPellegrino, GerardoCousins, JosephPellicano, RinaldoCoustau, ChristinePeng, Henry T.Coutinho, Maria FranciscaPeng, Xianlu LauraCowan, Kyra J.Perales, AlfredoCowden Dahl, KarenPeralta, Cleisson Fábio AndrioliCozzani, EmanuelePeran, IvanaCrawford, SeanPereira, BrunoCreytens, DavidPereira, FilipeCrino, Stefano FrancescoPereira, Maria A.Cristache, Corina MarilenaPereira, SofiaCrnkovic, AnaPérez-García, FelipeCroce’, Lory SaveriaPérez-Rodrigo, CarmenCronin-Golomb, MarkPerillo, LetiziaCrupi, VincenzaPerisetti, AbhilashCubiella, JoaquinPerlman, SharonCui, YanxiangPerls, Thomas T.Cuocolo, RenatoPero, RaffaelaCuric, GoranPeroz, IngridCymbaluk, AnetaPerri, FrancescoCzakó, LászlóPerrucci, Gianluca LorenzoCzekierdowski, ArturPerry, Anamarija M.Czimmer, JózsefPertinhez, ThelmaCzuczwar, MirosławPesce, AlessandroD’Agostino, DonatoPetejová, NadeždaD’Aguanno, SimonaPetera, JiriD’Argenio, ValeriaPetersen, Lars J.D’Arrigo, SoniaPetersen, SvenD’Elios, Mario MilcoPetersson, FredrikD’Orazi, ValerioPetrosino, MariaD’Orsi, BeatricePetrov, DmitryDąbrowska-Kugacka, AlicjaPfeiffer, David C.Dailey, George E.Piacenti, IlariaDaimi, HouriaPia̧tek, Rafał J.Dal Col, JessicaPiccin, AndreaDalessandri, DomenicoPiccinin, ElenaDandekar, AdityaPiccoli, MartinaDanilowicz-Szymanowicz, LudmilaPiciu, DoinaDarocha, Szymon DarochaPienar, CorinaDas, Bibhuti BhusanPierucci, PaolaDasí, FranciscoPignatti, PatriziaDavid, Vlad LaurentiuPilli, NageswaraDavis, Mellar P.Pilmane, MāraDawra, RajinderPincus, Matthew R.De Backer, WilfriedPinheiro, Joaquim M. B.De Baets, LiesbetPinho, Armando J.De Barrios, OriolPiorkowski, AdamDe Berardis, DomenicoPiotrowska, EwaDe Cobelli, OttavioPiotrowski, WojciechDe Falco, ValentinaPiovesan, AlessandroDe Franciscis, PasqualePirina, PietroDe Geus-Oei, Lioe-FeePirozzi, Christopher J.De Jonge, HugoPisapia, PasqualeDe La Luz Garcia-Hernandez, MariaPiskin, SenolDe Leo, AntonioPita, SebastianDe Mendoza, CarmenPittner, StefanDe Miguel, DiegoPiubelli, ChiaraDe Montpréville, VincentPiva, TerrenceDe Oliveira, Mario AndersonPluschke, GerdDe Pauw, JokePoddighe, DimitriDe Reijke, T. M.Podgorski, Iva I.De Reijke, Theo M.Poenar, DanielDe Ruiter, Godard C. W.Poetsch, AnsgarDe Sanctis, Juan BautistaPoirier, YannickDe Visser, MariannePolańczyk, AndrzejDe Zinis, Luca Oscar RedaelliPoldermans, DonDean, FrankPolewczyk, AnnaDeán-Ben, Xosé LuísPolguj, MichalDeandreis, DésiréePolitano, LuisaDe-Bandt, Jean-PascalPolkowski, WojciechDedhia, PriyaPollari, FrancescoDeftereos, GeorgiosPolovina, Snezana P.Deguelte, SophiePoltorak, LukaszDel Barrio, MelisaPomara, CristoforoDelacour, HervéPomari, ElenaDe-la-Cruz-Torres, BlancaPond, Amber L.De-los-Santos-Álvarez, NoemíPontikos, NikolasDelvaux, NicolasPonzetti, MarcoDelvecchio, MaurizioPonzoni, MaurilioDemyanets, SvitlanaPop, CristinaDepledge, DanielPop, Tudor LucianDerlin, ThorstenPopescu, Mihaela R.Deshpande, GauraviPoplawski, TomaszDeStefano, Christin B.Popovic, ZoyaDestrempes, FrançoisPoradowski, DominikDhama, KuldeepPorpora, Maria GraziaDi Agostino, SilviaPorro, ChiaraDi Bella, GianlucaPortelli, MarcoDi Diego, JosePósa, AnikóDi Domenico, GiovanniPourlis, ArisDi Donato, MarziaPowell, Folami LamokeDi Gennaro, FrancescoPowrózek, TomaszDi Girolamo, StefanoPrakash, KN BhanuDi Lorenzo, FrancescoPrasad, VikasDi Luciano, CarmelaPravdin, SergeiDi Profio, FedericaPrchal, Josef T.Di Spiezio Sardo, AttilioPribilova, AnnaDi Stasio, EnricoPrincipia, Scavo MariaDi Tommaso, LuigiPrintza, NikoletaDiamantopoulos, AndreasProdromidou, AnastasiaDias, FranciscaProtonotarios, AlexandrosDidona, DarioPruna, DarioDieks, Jana-KatharinaPrzybylik-Mazurek, ElwiraDietrich, CharlesPrzybyło, MałgorzataDinets, Andrii V.Pshezhetsky, AlexeyDionne, AudreyPsuj, GrzegorzDiorio, CarolinePucci, MairiDir, JanPugliese, Nicola RiccardoDittmann, HelmutPuglisi, FabioDivjak, EugenPugni, LorenzaDłuski, DominikPulavarti, Surya V. S. R. K.Dmochowski, MarianPulford, KarenDo, Minh TruongPulito, ClaudioDocea, Anca OanaPuolakkainen, MirjaDocimo, LudovicoPuri, KritiDoddapattar, PrakashPuthenparampil, MarcoDomanin, MaurizioPuzzovivo, AgataDomenici, GiacomoQadura, MohammadDonati, MarcelloQasim, MuhammadDong, QinglinQi, Qian-RongDonnelly, Callum GeorgeQian, XuejunDorman, DavidQiu, Jiantai TimothyDosoky, NouraQiu, ZhenDou, LaetitiaQuattrone, AndreaDoulberis, MichaelQue, JennyDresselaers, TomQueiro, RubénDriessen, Gertjan J.Quiróz-Mercado, HugoDrummond, Gordon B.Rabkin, Simon W.Druszczynska, MagdalenaRabuazzo, Agata MariaDu, KeRadzikowska, ElzbietaDubrova, YuriRaeeszadeh-Sarmazdeh, MaryamDucarme, GuillaumeRafi, JunaidDuchene, BernardRagavan, MukundanDunkley, Benjamin T.Ragazzoni, AldoDunowska, MagdalenaRahelić, DarioDuong, PhuRahman, HafizurDuong-Quy, SyRahman, Masmudur M.Duraturo, FrancescaRahman, QuaziDurazzo, AlessandraRahman, SafikurDutta, AniruddhaRaikhy, GauravDuzgunes, NejatRaimondi, LaviniaDzięgiel, PiotrRaimondo, DiegoDziurkowska, EwelinaRainieri, EnzoEboigbodin, KevinRaizman, RoseEcke, Thorsten HolgerRajda, CecíliaEdelman, BradleyRaju, NagarajanEdlefsen, Paul T.Ramamurthy, NitinEeckhoute, JérômeRamasamy, RanjanEgenlauf, BenjaminRamos, CarmenEhrenpreis, Eli D.Rampanelli, ElenaEid, NabilRamprasath, TharmarajanEiring, AnnaRana, JyotiEkpo, Ernest U.Rapado-González, ÓscarEl Hadi, HamzaRapi, StefanoEl Midaoui, AdilRapisarda, Agnese Maria ChiaraElkins, KellyRapone, BiagioEllmann, StephanRäsänen, EsaElsayad, KhaledRashid, Mohammad HarunEl-Sheikh Ali, HossamRastogi, MadhupEndo, MakotoRatiu, AndreeaEnninga, Elizabeth Ann L.Rattray, ZahraErdmann, KatiRaveglia, FedericoEreminienė, EglėRaya, AnaEriksson Steigen, SonjaRaz, EytanErovic, Boban M.Razvan-Cosmin, PetcaEscudero-Pérez, BeatrizRebelo, SandraEsfahani, SiavashReddy, SakamuriEspa, StefaniaReddy, Sudhir PuttyEsworthy, Robert StevenRedente, Elizabeth F.Eun, Jung WooRega, DanielaEvangeliou, AthanasiosReichenbach, Zachary WilmerEvans, Catherine J.Ren, JunEvans, Dafydd GarethRen, PingExbrayat, Jean-MarieRengo, MarcoExindari, MariaRenz, DianeEykyn, ThomasRenzini, AlessandraEzhov, MaratRequena, ManuelFabien, ForestRestaino, StefanoFacciorusso, AntonioReyes-Aldasoro, Constantino CarlosFadda, GianlucaRezania, ShahabaldinFadda, GuidoRezus, CiprianFaggioni, LorenzoRibaldone, DavideFagioli, FrancaRibeiro, RicardoFalzone, LucaRibera, JordiFan, Hueng-ChuenRicardo, RiberioFanizzi, AnnaritaRiccardo, ZojaFanti, AlessandroRicchiuto, PieroFarina, NicholasRichter, WitoFaron, AntonRiechelmann, HerbertFarrell, Philip M.Riethmüller, ChristophFarschtschi, Said ChosroRiis, Margit L. H.Fatima, IramRiou, Laurent M.Faustino, AnaRipa, Rasmus SejerstenFedele, FrancescoRizzello, FrancescaFederico, RavegliaRizzo, CristianoFehr, AndréRizzo, StefaniaFeier, Diana SorinaRoberts, Drucilla J.Feier, HoreaRoby, Justin A.Feng, HuichengRocchi, AnnaFeng, WanjuanRocco, DaniloFeodorova, Valentina A.Roceanu, AdinaFeregrino-Pérez, Ana A.Rochani, AnkitFernandes Fagundes, NathaliaRochtus, Anne M.Fernández De Las Peñas, CésarRočka, SauliusFernandez, PatriciaRodrigo, Juan P.Fernández-Lázaro, DiegoRodrigo-Muñoz, José M.Fernandez-Matias, RubenRodrigues-de-Souza, Daiana PriscilaFernando, WyssRodríguez Hermosa, Juan LuisFerrara, FrancescoRodríguez, William R.Ferrara, MariantoniaRodríguez-Almagro, DanielFerrara, PietroRodríguez-Sanz, DavidFerrarazzo, GiuliaRoedel, FranzFerrari, CristinaRogobete, Alexandru FlorinFerrari, FedericoRoje, DamirFerreira, FernandoRojo, Maria AngelesFerrero, SimoneRola, RadosławFerri, FlaminiaRolla, GiovanniFerro, MatteoRomano, LorenzoFilho, Julio RicarteRomero, Marta R.Filip, Stanislav S.Romero-Castro, RafaelFilippiadis, Dimitrios K.Ronen, OhadFilippiadis, DimitrisRong, Chan KuanFinelli, CarmineRooney, ClaireFineschi, VittorioRosado, CarlosFiorillo, LucaRosario, BarrancoFisse, Anna LenaRosca, MonicaFiz, FrancescoRose, Christopher F.Flores, IdhalizRoss, RyanFlores-Guerrero, Jose L.Rossi, FrancescaFloria, MarianaRossi, GemmaFochi, StefaniaRossi, RiccardoFoecking, MelanieRossitto, GiacomoFoguel, Marcos ViniciusRossow, Lindy M.Font, AlbertRostoker, GuyForest, FabienRoubalova, KaterinaForget, PatriceRoumiguie, MathieuForlano, RobertaRovas, LinasForster, JamesonRovina, DavideForte, MaurizioRovnak, JoelFosbøl, Marie ØbroRowley, K. MichaelFoster, Kenneth R.Roy, DhruvajyotiFoti, Daniela P.Rozerta, SokouFourati, SlimRubegni, PietroFrame, Leigh A.Rubello, DomenicoFrancesco, NudiRudroff, ThorstenFrancis, Ian C.Rundo, LeonardoFranza, LauraRuscitti, PieroFrati, PaolaRussell, DarrenFrauenfelder, ThomasRusso, AlessandroFredriksson, IngemarRusso, AlessiaFriedmacher, FlorianRusso, GiorgioFrietze, SethRusy, Lynn M.Frigy, AttilaRutkauskas, SauliusFritz, JanRužauskas, ModestasFroelich, Matthias F.Ruzzini, LauraFrulloni, LucaRychtáriková, RenataFu, Pin-KueiRyozawa, ShomeiFujioka, TomoyukiRyswyk, Emer VanFujishima, HiroshiSabatino, GiovanniFujita, ToshitsuguSabiniewicz, RobertFukuda, AkiraSaccardi, CarloFukui, AtsushiSachar, David B.Fukumoto, YoshihiroSachithanandan, NirupaFukumura, YukiSachpekidis, ChristosFukushima, HiroshiSaczonek, Agnieszka OwczarczykGabel, KelseySadahira, TakuyaGabelloni, MichelaSadasivam, MohanrajGach, AgnieszkaSahoo, PrativaGadda, GiacomoSahoo, SubhransuGaddi, Antonio VittorinoSahrmann, PhilippGaffney, ChristopherSaigusa, DaisukeGagliardo, CesareSaijo, YoshifumiGagniuc, PaulSaini, ShikhaGajewska, DanutaSakai, FumikazuGalan Mercant, AlejandroSakamoto, RyuichiGalani, Vasiliki A.Sakamoto, ShinichiGalán-Sánchez, FátimaSakthivel, KogularasuGalati, FrancescaSakuma, KunihiroGalinetto, PietroSalagierski, MaciejGall Troselj, KoraljkaSalehzadeh-Yazdi, AliGallagher, Philip M.Salerno, SergioGallego-Colon, Enrique J.Salido Tercero, JesúsGalvosas, PetrikSalomone, AlbertoGama, AdelinaSalvador, PabloGambini, JuanSalvi, MassimoGanau, MarioSam, RaminGancarz, MarekSánchez-Ávila, Ronald MauricioGandham, AnoohyaSánchez-Bueno, FranciscoGao, Xiaoyi RaymondSánchez-Gómez, RubénGarbers, ChristophSánchez-González, José-MaríaGarcía, MontserratSancisi, ValentinaGarcía-Vicente, Ana MaríaSanders, MatthewGarg, BhavukSandwall, PeterGarinis, GeorgeSanguedolce, FrancescaGascho, DominicSantander, PetraGąsior-Perczak, DanutaSantarelli, AndreaGaspar, PhyllisSanthanam, AbiramiGasperini, SerenaSantoro, ClaudiaGaur, NishthaSantos, João H. P. M.Gaviria, ManuelSantos, RuteGawel, DamianSantosh, K. C.Ge, XiaodongSantucci, AnnalisaGeddes-McAlister, JenniferSantulli, GaetanoGeisler, RamsiaSanz, AlejandroGelati, MatteoSaravanakumar, KandasamyGelb, MichaelSardu, CelestinoGelsomino, SandroSarkar, AmritaGentili, FrancescoSarria-Santamera, AntonioGentilin, EricaSasaki, EijiGeorgiev, Georgi AsSasase, TomohikoGerke, OkeSato, AkiraGermana, AntoninoSato, EiichiGervasi, RitaSato, Takashi Shawn P.Ghanbarzadeh-Dagheyan, AshkanSavchenko, Andrey A.Ghandour, RashedSavoia, PaolaGherman, BogdanSawicki, MarcinGhidini, MicheleSaynor, ZoeGhio, StefanoSazonova, Margarita A.Ghiroldi, AndreaSbrollini, AgneseGiammarile, FrancescoScacco, SalvatoreGiampieri, RiccardoScarano, AntonioGianfredi, VincenzaScaravilli, VittorioGiangregorio, FrancescoScarlata, SimoneGiannakopoulos, PanteleimonScelsa, BarbaraGiannella, LucaSchäfer, Valentin S.Giannone, GaiaSchares, GereonGiannotti, ElisabettaScharffenberg, MartinGianturco, LuigiSchell, MichaelGibbs, PeterSchepisi, GiuseppeGibson, James B.Schiattarella, AntonioGiebultowicz, JoannaSchiavone, MarcoGilmour, Susan M.Schiff, Elena R.Gioia, SaraSchiffer, EricGiordano, GuidoSchildberg, Frank A.Giordano, RaffaeleSchillaco, OrazioGiordano, SalvatoreSchiller, AdalbertGiotakis, Aris Ι.Schirinzi, TommasoGioulekas, FotiosSchmidl, DoreenGiovagnoli, RaffaelaSchmitz, RalfGiovannelli, PiaSchmoll, DieterGiraudo, ChiaraSchmutz, AxelGiri, HemantSchoenhagen, PaulGironella, MeritxellSchott, UlfGish, RobertSchreiber, StefanieGiudice, Giuseppe LoSchrier, RachelGiuffrè, MauroSchröder, AgnesGiungato, PasqualeSchuetz, GunnarGladysheva, Inna P.Schulz, Wolfgang A.Glaudemans, AndorSchwind, SebastianGlowacz, AdamScimeca, ManuelGlushakov, AlexanderScisciola, LuciaGnanasundram, Sivakumar VadivelSeghaye, Marie-ChristineGodde, KanyaSehgal, Chandra M.Goel, PratibhaSen, SougataGoergen, Craig J.Senanayake, SameeraGoldhawk, Donna E.Sensi, MatteoGoldis, AdrianSeong, Jin-TaekGołębiowski, TomaszSerafín, VerónicaGolubovskaya, VitaSerafini, GianlucaGómez-Barrero, SusanaSeravalle, GinoGonzalez, GabrielSerban, Daniela ElenaGonzalez, GermanSergi, ConsolatoGonzalez, RogelioSério, SusanaGonzalez-Penas, JavierSerpico, RosarioGorczynski, AdamSerry, MohamedGordeeva, OlgaSevvana, MadhumatiGori, TommasoSeydel, KarlGormsen, Lars C.Shackelford, Rodney E.Gorrell, MarkShah, JainilGoto, KatsumasaShah, NeerajGoto, TaichiroShahzad, AtifGovindarajan, Sindhuja TirumalaiShamshirband, ShahabGoździewska-Harłajczuk, KarolinaShan, HongmingGrabarek, Beniamin OskarShankar, EswarGrabmüller, MelanieShao, WeiGrady, Erin E.Shapiro, AdrienneGraham, SheilaSharara, FadyGrant, GeorgeSharker, Shazid Md.Grasso, GiuseppeSharma, GauravGray, Michael D.Sharma, NishaGraziani, MaristellaSharon, DeniseGreim, HelmutShaw, Alyra J.Greuter, MarcelShaw, George J.Grimm, AlexanderSheikh, AlaullahGrimm, Marcus O. W.Shen, LimingGris, DenisShen, Ming-YuanGrizzi, FabioShen, ShichenGrochowski, CezarySheu, JimGroeneveld, DafnaShi, JunchaoGrošelj, UrhShi, LeiGrosse, Scott D.Shibukawa, GoroGrymowicz, MonikaShih, DavidGrzanka, DariuszShih, Ie-MingGu, BinShiina, MarisaGuadagni, StefanoShimada, YohtaGuagnano, Maria TeresaShimatani, MasaakiGuarnieri, GabriellaShimizu, YujiGuembe, MariaShinde, AparnaGuenancia, CharlesShintaku, HaruoGuerriero, StefanoShirai, JyunsukeGuglielmi, ValeriaShirshin, EvgenyGuglielmo, PriscillaShivakumar, PranavGuillaud, MartialShoji, SunaoGulei, DianaShokoohi, HamidGulino, Ferdinando AntonioShukla, KirtikarGuo, YanhuiSiddiqui, ImranGupta, KuldeepSidorov, Denis NikolaiGupta, MohitSidorov, EvgenyGupta, VijayalaxmiSier, CornelisGursoy, MerviSignore, AlbertoGutte, HenrikSiiskonen, TeemuGyselaers, WilfriedSijens, Paul E.Habanova, MartaSikalidis, AngelosHabiba, MarwanSikora, MariuszHafer, CarstenSilva, CarlosHaider, NomanSilva, CatarinaHaijes, HannekeSilva, Marcelo SousaHaj-Mirzaian, AryaSilva, Susana NunesHall, KateSimakov, SergeyHallac, RamiSimka, MarianHalloran, KieranSimko, FedorHalmosi, RóbertSimmonds, RachelHalsall, David J.Simoes, JoanaHam, Won-sikSimon, ThomasHamaguchi, ShogoSimonov, NikolaiHamedpour, VahidSimopoulou, MaraHamm, RobertŠindlerová, LenkaHan, Der-ShengSingh, AmritaHanai, Jun-ichiSingh, KamayaniHanas, JaySingh, Pankaj K.Hansen, Søren BaarsgaardSingh, PratibhaHanssens, LaurenceSingh, SandeepHapuarachchi, Hapuarachchige ChandithaSingh, SukhvinderHardwick, James P.Singh, Sumit RandhirHargreaves, IainSinghal, TarunHarris-Love, Michael O.Sinha, NiharikaHartmann, JanSinning, ChristophHassaballah, M.Sireci, FedericoHatzl, StefanSisti, GiovanniHavre, Roald FleslandSjödahl, GottfridHawkins, FedericoSkoczyński, SzymonHeaney, J. L.Škrlec, IvanaHedfalk, KristinaSleigh, JamesHedman, KlausSlezák, RadovanHedriana, Herman LocsinSliwinska-Mosson, MariolaHeise, BettinaSloan, KennethHeitmar, RebekkaŚmigiel, RobertHelm, Matthew F.Smith, David F.Heng, XiaoSmith, Gary A.Hénique, CaroleSmith, Heather F.Hennerici, Michael G.Smith, Lloyd M.Henrique, RuiSmyczyńska, JoannaHepojoki, SatuSnyder, Christopher S.Hernandez, FrankSobhani, NavidHernández, SusanaSobolewski, EricHernandez-Vaquero, DanielSocea, BogdanHernes, MarcinSochaj-Gregorczyk, Alicja MariaHervás Pérez, Juan PabloSohns, Jan Martin SommerlathHervouet, EricSoica, CodrutaHerynek, VítSolé Cañadas, CarlaHida, RichardSolé, CristinaHikichi, TakutoSoler, CarlesHildebrandt, Malene GrubbeSolimando, Antonio GiovanniHildreth, BlakeSolís-Lemus, José AlonsoHilhorst, MarcSolitro, GiovanniHingorani, DinaSomboonwit, CharurutHingorani, RittuSommariva, ElenaHippensteel, JosephSong, Chang MyeonHirai, KeitaSong, In-SeokHirano, TsunahikoSorrentino, Francesco SaverioHirata, KenjiSorsa, TimoHlaváč, ViktorSotnikov, Dmitriy V.Ho, ChichunSpagnolo, Elvira VenturaHo, Shih-ChingSpalice, AlbertoHobolth, LiseSpang, ChristophHoeschen, ChristophSpano, GiuseppeHohaus, StefanSpanu, AngelaHokamp, Nils GroBeSpartalis, EleftheriosHołda, Mateusz KrystianSperanza, DomenicoHöllig, AnkeSperti, CosimoHolobar, AlešSpong, CatherineHolzinger, AndreasSpooner, NeilHoriguchi, TakashiSporea, IoanHörmann, GregorSprinkart, Alois M.Horne, MalcolmSreedharan, SreejeshHorsburgh, AnnSridhar, SubbaramiahHorwath-Winter, JuttaStaboulidou, IsminiHosohata, KeikoStacey, Michael C.Hostiuc, SorinŠtajduhar, IvanHöti, NaseruddinStanciu, Gabriela-DumitritaHou, YanjunStanculeanu, Dana LuciaHoude, PeterStandley, Claire J.Hough, KennethStanley, Jone A.Howard, Brittany E.Starshinova, Anna AndreevnaHsiao, Kuei-YangStawski, RobertHsiao, Sheng-MouStecco, AlessandroHsiao, Yi-HsuanStefanidis, Constantinos J.Hsieh, Ming-ChingStefanini, LuciaHsieh, Tsung-HanStefano, AlessandroHsieh, Tusty-JuanStefanovic, IvanHsu, Bang-geeStefanowicz, JoannaHsu, Chung-YuanSteibliene, VestaHsu, Chun-NanSteinbichler, Teresa BernadetteHsu, Li-YuehSteponaitis, GiedriusHsu, Yi-ChiungStepp, Mary AnnHu, BaoliSteyger, PeterHu, TaoboStiglic, GregorHu, YingStillo, FrancescoHuang, Chih-ChungStoffman, JaysonHuang, HuachaoStoian, DanaHuang, MarilynStonbraker, SamanthaHuang, Sarah XuelianStrati, AretiHuang, Tzu-TingStraudi, SofiaHuang, XuStrekalova, TatyanaHuang, Yao-KuangStruck, ManuelHudlikar, RasikaSturiale, Carmelo LucioHueso, MiguelStutz, BernardoHughes, Sean P. F.Su, Chih-MinHund, MartinSu, Kuo-ChihHung, Kuo-ChuanSubedi, DineshHuss, AndreSubhi, YousifHwang, Eun SeongSuciu, Bogdan AndreiHwang, Jae YounSuga, JenniferHwang, Sung HoSugiura, TsuyoshiHwang, Yi-TingSuhail, YasirIacopi, ElisabettaSujit, Sheeba JeniferIacoviello, MassimoSukocheva, OlgaIasevoli, FeliceSulikowski, PiotrIbrahim, Sherif AbdelazizSumi, YasuoIde, KazukiSun, DaekyuIdicula, Titto T.Sun, LiIentile, RiccardoSun, ZhixiongIeraci, AlessandroSun, ZhonghuaIgnat, AncaSung, Wen-WeiIjare, Omkar B.Surani, SalimIlardi, FedericaSuraweera, AmilaIles, RaySutoh, YoichiIlhan, HarunSuzuki, MotofumiIlias, IoannisSuzuki, ShugoIllert, LenaSuzuki, YoriyasuIm, Hyung-JunSwami, UmangIm, HyungsoonSyafrudin, MuhammadImamura, TeruhikoSyeda-Mahmood, Tanveer FathimaImbimbo, BrunoSzabó, DóraInbar-Feigenberg, MichalSzczepanek, JoannaIndraccolo, UgoSzlubowski, ArturInglada-Pérez, LucíaSzoboszlai, NorbertIngravallo, GiuseppeSzopa, AndrzejInnaro, NadiaSzubert, SebastianInomata, MinehikoSzutkowski, KosmaInoue, KazushiTaffé, PatrickInoue, TakamitsuTagliabracci, AdrianoInusa, BabaTajika, TsuyoshiInzani, FredianoTajiri, NaokiIqbal, Hafiz M. N.Takabatake, KiyofumiIravani, AmirTakabayashi, ShujiIrie, DaisukeTakač, IztokIrisawa, AtsushiTakado, YuheiIrzyniec, TomaszTakaki, ManabuIsayama, HiroyukiTakamatsu, ShigeyukiIsaza Martínez, José HipólitoTakebayashi, ShigeoIshikawa, TomohiroTaked, TakayukiIskander, RobertTakeda, HiroyukiIsola, GaetanoTakedatsu, HidetoshiIstván, HornyákTakeishi, NaokiItenov, Theis SkovsgaardTakeuchi, RikiyaIto, TakamichiTalaat, Iman M.Itonaga, MasahiroTallmadge, RebeccaIwata, JunichiTamaki, NobuharuIyer, JanakiTan, KarenIzake, Emad L.Tan, NingIzawa, Kazuhiro P.Tan, WenbinIzonin, IvanTanaka, NaoroIzumi, GentaroTanaka, TakashiIzumi, KoujiTang, FangfangJabłonowski, ZbigniewTangaro, SoniaJabłońska, BeataTanner, NathanJacob, NoamTanyeri, MelikhanJafari, JafarTao, Jeremiah P.Jagodzinski, Linda L.Taradaj, JakubJahrami, HaithamTarantino, GiovanniJaidev Chakka, Leela RaghavaTashima, ToshihikoJaime, Diego FrancoTavares, João Manuel R. S.Jakobsen, Marianne A.Tayek, John A.Jalan-Sakrikar, NidhiTaylor, AndyJambor, IvanTaylor, MatthewJanakiraman, HarinarayananTeague, Shawn D.Jang, Sung HoTedaldi, GianlucaJankauskiené, AugustinaTeixidó, CristinaJans, Judith J. M.Teng, Ru-JengJanzen, NilsTeragawa, HirokiJaouen, VincentTerayama, HayatoJape, GayatriTerracciano, DanielaJarosz-Chobot, PrzemysławaTesarik, JanJårvinen, Tero A. H.Teteloshvili, NatoJastrzebska, AgnieszkaTeus, MiguelJeannot, EmilienTha, Khin KhinJeerapan, ItthiponThakur, Sunitha N.Jenssen, ChristianThan, Nandor GaborJeong, Hwal RimThekkinkattil, Dinesh K.Jeong, Keun-YeongThevenot, Paul T.Jerebtsova, MarinaThior, IbouJeremy, Teoh Yuen ChunThirunavukkarasu, ShyamalaJeruszka-Bielak, MartaThomsen, ThomasJeschke, UdoThomssen, ChristophJežek, JanThornell, Ian M.Jezela-Stanek, A.Tian, FurongJezierski, TadeuszTian, WeihuaJiang, ChunjieTimchenko, PavelJiang, HuaileiTimchenko, TatianaJiang, JianxiongTimmons, Mark K.Jiang, XuezhiTinelli, AndreaJiao, ZhichengTito, Raúl YhossefJimenez Garcia, Victoria AlejandraTjioe, MarcoJing, RanTjong, Sie ChinJochelson, MaxineTkáč, JánJochum, ChristophTodsen, TobiasJødal, LarsToelle, BrettJohansson, Mats W.Toews, MatthewJohns, Terrance G.Togawa, YaeiJohnson, Jason MichaelTokarek, TomaszJones, MarkTokas, TheodorosJönsson, DanielTolmachev, VladimirJoyner, Chester J.Tomatsu, ShunjiJu, Wei-JhongTomczyk, ArkadiuszJuhasz, BelaTomescu, Mirela CleopatraJuhasz, CsabaTomsone, SigneJung Bae, YunTonet, ElisabettaJung, KlausTong, Yat-ChingJung, SungmokTonni, GabrieleJunglee, Naushad AliToriello, FilippoJunker, KlausTörök, PeterKachlik, DavidTorras-Ambròs, JoanKaden, MarikaTorres, JorgeKaese, SvenTorres-Sangiao, EvaKaewput, WisitToshchakov, StepanKagawa, MasaharuTosi, DavideKagiyama, NobuyukiTosun, DuyguKaigorodova, EvgeniyaToth, KalmanKaindl, AngelaTouil-Boukoffa, ChafiaKajtár, BélaTovoli, FrancescoKakkassery, VinodhToyos, MelissaKakoti, AnkanaTozzo, PamelaKalef-Ezra, John A.Trainor, PatrickKalinin, AlexandrTrecroci, AthosKallner, AndersTrendelenburg, MartenKalogianni, DespinaTreviño, MercedesKalogirou, CharisTrimarchi, MatteoKamata, KenTrimboli, PierpaoloKaminski, RafalTrinci, MargheritaKamolz, Lars P.Tripodo, ClaudioKan, Chung-DannTriunfo, StefaniaKang, JeonghyunTrouillas, JacquelineKant, SashiTrubiani, OrianaKanyong, ProsperTrudel, DominiqueKapischke, MatthiasTsai, JamesKapočiūtė-Dzikienė, JurgitaTsai, Kun-LinKarayiannakis, AnastasiosTsai, RongkungKarbownik-Lewińska, MałgorzataTsakanikas, PanagiotisKarhula, SakariTsang, Kwong YokKarlinger, KingaTsangaris, IraklisKarlsson, JoakimTsoi, James Kit-honKarmazyn, MorrisTsubota, AkihitoKaroor, VijayaTsuchiya, JunichiKarpiński, Tomasz M.Tsuchiya, TakayoshiKarshikoff, BiankaTsunematsu, TakaakiKarteris, EmmanouilTu, Hung-PinKarvouniaris, MariosTuovinen, TeroKarvounis, ArtemiosTuriel, MaurizioKashiwagi, ShinichiroTurner, JonathanKashtalap, VasilyTurner, RichardKasprzak, JaroslawTutino, Vincent M.Katagiri, HiroshiTuttolomondo, AntoninoKataoka, MasakoTwarock, SörenKatayama, TakayukiTyan, Yu-ChangKate, MaheshTyler, NicholeKato, KatsuhikoTzortzakakis, AntoniosKaufmann, Walter E.Tzschatzsch, HeikoKauppinen, AnuTzur, TamarKaushik, AniruddhaUchiyama, KojiKawabata, HideakiUeda, KojiKawanami, DaijiUgga, LorenzoKawano, KouichiroUgon, AdrienKawasaki, HideyaUlivi, PaolaKazui, ToshinobuUllah, AminKelcz, FrederickUllah, IrfanKeller, BrandisUmano, Giuseppina RosariaKellett, John G.Umapathy, GaneshKelley, ThomasUmemori, JuzohKerman, KaganUmemura, AtsushiKesmarky, GaborUmutlu, LaleKessler, Ronald C.Unchwaniwala, NuruddinKhalek Abdel Razek, Ahmed AbdelUngurianu, AncaKhan, AmjadUpadhyaya, JasbirKhan, KrishnenduUrbano, NicolettaKharche, SanjayUrbanska, Edyta MariaKhodanovich, MarinaUribe, Alberto A.Khokhar, BilalUsai Satta, PaoloKholmukhamedov, AndalebUsui, NoriyoshiKholová, IvanaUtsunomiya, DaisukeKhushi, MatloobUyar, Denise S.Kiesewetter, Dale O.Vaculovičová, MarketaKikuchi, ShogoVafaee, ManouchehrKikuyama, MasatakaVaibhav, KumarKim, Beom KyungVaira, ValentinaKim, BongleeValentinčič, Nataša VidovičKim, Dal YoungValenzuela-fernández, AgustínKim, HyungjooValverde, Jose R.Kim, Hyun-SooVan Bussel, Bas C. T.Kim, Jee TaekVan Den Berge, MargreetKim, Jin SuVan Den Meiracker, Anton H.Kim, Jin-wooVan Der Burgt, InekeKim, JungyoonVan Etten, Ellis S.Kim, Raymond H.Van Hal, SebastiaanKim, Ryong NamVan Heerden, Peter VernonKim, SeonghoVan Rhee, FritsKim, SeongjaeVan Timmeren, JanitaKim, Seong-TaeVan Trijffel, EmielKim, Tae HyunVancheri, FedericoKim, YerimVandenbriele, ChristopheKim, Yeun-YoonVanzi, FrancescoKim, Yong JinVardhanabhuti, VarutKimber-Trojnar, ŻanetaVarela, GonzaloKinfe, Thomas M.Varela-Nieto, IsabelKinkorová, JuditaVasefi, MaryamKirac, IvaVashist, Sandeep KumarKisand, KaiVaskelyte, Jolanta-JustinaKishaba, TomooVassiliou, Aliki GeorgiaKiss, RitaVaughan, Christopher L.Kistenev, YuriiVaverka, MiroslavKitrungrotsakul, TitinuntVaz, PedroKittl, SonjaVecchio, MicheleKlapper, Stephen R.Veenland, JifkeKlar, MaximilianVegas-Sánchez-Ferrero, GonzaloKlatt, DieterVeiga, Maria IsabelKlebe, SonjaVeilleux, Louis-NicolasKleczynski, PawelVelan, SendhilKlobučar, HrvojeVeldeman, MichaelKlymenko, YuliyaVelikova, TsvetelinaKnezevic, JelenaVenkataraman, RajeshKnudsen, Jonas R.Venkatesh, Alagarswamy G.Koch, AchimVenkateswaran, MahimaKoga, FumitakaVentura, PaoloKoivusalo, Antti IlmariVenturella, RobertaKojima, TakashiVersari, AnnibaleKolberg, Hans-ChristianVidili, GianpaoloKolesar, Jill MarieVieira, Edgar RamosKolonko, AureliuszViggiano, AndreaKoltz, Michael T.Viggiano, DavideKomuraiah, MyakalaVilahur, GemmaKondru, NaveenVillani, RosannaKones, RichardVincent, Amy E.Kong, Kien VoonVincenzi, ColombinaKonishi, MasaakiVirella, DanielKonoike, NahoVirumbrales-Muñoz, MaríaKontos, Christos K.Visentin, SilviaKoo, Kyo ChulVitale, GiuseppeKoolivand, AbdollahVitale, Salvatore GiovanniKooyman, David L.Vitoratou, SiliaKopechek, JonathanVitorino, Rui Miguel PinheiroKoper-Lenkiewicz, Olga MartynaVlamos, PanayiotisKopyta, IlonaVlk, MartinKoshiyama, MasafumiVo, DatKosiak, WojciechVoellger, BenjaminKosmala, AleksanderVogel, Wouter V.Kosmas, PanosVoiculescu, DanKost, GeraldVolaklis, KonstantinosKostkiewicz, MagdalenaVolkau, IharKostrzewa-Nowak, DorotaVon Arx, ClaudiaKosuke, MatsubaraVon Blanckenburg, PiaKoszelewski, DominikVora, MehulKotani, ToruVos, CornelisKothapalli, Sri-RajasekharVrachatis, Dimitrios A.Kotter, ElmarVriens, DennisKotyla, Przemyslaw J.Vujović, IgorKourou, KonstantinaWagner, Kay-DietrichKovács, ÁrpádWakabayashi, HiroshiKovács, Árpád FerencWalocha, JerzyKoyama, EikiWan, ShibiaoKoyama, TakashiWang, BinKraaijeveld, Adriaan O.Wang, CemingKramvis, AnnaWang, Chen-YuKrasiński, ZbigniewWang, Chun-HouKrawiec, PaulinaWang, DongwenKrazinski, Bartlomiej EmilWang, DongyiKreimer, SimionWang, Hsiang-ChenKreiser, KorneliaWang, HsiuyingKrewer, CarmenWang, HuanyuKrick, StefanieWang, KaiKriegova, EvaWang, MaosenKrishna, Somashekar G.Wang, MengyuKristensen, GitteWang, Shao-HungKroiss, AlexanderWang, Tsung-JenKrupa-Kozak, UrszulaWang, WilliamKrzych, LukaszWang, XueKrzyżak, Artur TadeuszWang, Yeng-TsengKrzyżanowski, ArkadiuszWang, YiKrzyżewski, RogerWang, YixiangKshirsagar, Prakash G.Wang, YumengKubera, MartaWang, ZhanxiangKubo, EriWang, ZhaoningKuboto, KazuoWansbrough-Jones, MarkKuddus, Ruhul H.Ward, MarkKuessel, LorenzWarsof, Steven L.Kuhn, ElisabettaWarzecha, ZygmuntKulczyk, TomaszWashington, Anthony ValanceKullaa, ArjaWatabe, TadashiKumar, BrajeshWaterhouse, MiguelKumar, GauravWatier, BrunoKumar, SuneelWatson, DesKumaraswamy, ChitralaWawruszak, AnnaKumari, SonamWeaver, Kathryn NicoleKummer, KaiWebster, BonnieKung, Hank F.Webster, DianneKunicki, MichałWeglarz, WladyslawKunz, WolframWegrzyn, GrzegorzKuo, Ming-TseWehrhahn, MichaelKurasova, OlgaWei, Wei-ChyouKurata, AkiraWeiner, JulianeKuriakose, MajuWells, JackKurir, Tina TičinovićWełnicki, MarcinKurzyna, MarcinWerner, EricaKushima, MikiWesthoff, NiklasKushkevych, IvanWetz, ChristophKusunose, KenyaWhiteley, Mark S.Kutikhin, Anton G.Widera, MarekKutsumi, HiromuWiderska-Chadaj, Zaneta S.Kuwabara, YoshihiroWiechowska-Kozłowska, AnnaKuwahara, TakamichiWiehe, ArnoKwak, HyoSungWielgos, MiroslawKwon, Seong JungWietrzyk, JoannaKyo, SatoruWilczek, PiotrLa Forgia, DanieleWilkinson, NafisaLa Fougère, ChristianWilliams, E. MarkLa Gatta, AnnalisaWilson, Alphus D.La Mura, VincenzoWinn, BryanLa Rosa, Valentina LuciaWitczak, Carol A.La Vignera, SandroWitowski, JanLabrou, NikolaosWojsyk – Banaszak, IrenaLabrune, PhilippeWolfram, FrankLachmann, RobertWong, Kwong-KwokLacina, LukasWong, MauriceLaganà, Antonio SimoneWong, Season S.Lahooti, HooshangWong, Yon-CheongLai, KentWoo, SungminLai, XinWorrell, StephanieLaihia, JarmoWoźniak, MateuszLajolo, CarloWozniak-Knopp, GordanaLam, Wai-ChingWróblewska-Seniuk, KatarzynaLam, WilburWrona, EwaLamango, Nazarius S.Wu, (Jason) BoyangLambrou, George I.Wu, Chau-ChungLançon, ChristopheWu, Chiao-EnLandberg, EvaWu, Chueh-HungLandegger, Lukas D.Wu, Chun-HuLang, DiWu, Hsien-TsaiLang, Hans PeterWu, Wei-TingLang, MichaelWujtewicz, MagdalenaLang, Nikolaus WilhelmWysocki, MarianLanza, MicheleXiang, HandanŁapaj, ŁukaszXiao, PerryLapini, AlbertoXie, JiexiongLaptoiu, DanXie, LingxiLarin, KirillXie, WankunLarsen, Anders ChristianXie, YibinLatosinska, AgnieszkaXu, ChenchuLattanzi, RiccardoXu, GuanLaura, EvangelistaXu, GuofangLavdaniti, MariaXu, JinbinLazăr, Daniela CorneliaXu, PengLazzarotto, TizianaXu, RongLe Page, CécileXu, Wen TaoLe, Thi-Thu-HuongXu, XiaowenLebogang, LesediYadav, DhananjayLee, Alvin J. X.Yagmur, ErayLee, Byung KookYahyaoui, RaquelLee, ChanghoYamada, HiroyukiLee, Chia-HwaYamada, ShuheiLee, Chii-MingYamada, YukioLee, Chu-YuYamaguchi, SatoshiLee, Dong YunYamasaki, TriciaLee, HeedooYamauchi, AkiraLee, Hsiang-ChiehYan, KeLee, Jeong WonYan, ShengminLee, Jong YunYan, XuefengLee, Joo HyoungYang, Chih-JenLee, JuhyunYang, Chih-YuLee, KijoonYang, Chun ZhangLee, Ki-SunYang, HaoLee, Kyung-YilYang, Joshua Y. C.Lee, Lukas Jyuhn-HsiamYang, Kuen-ChehLee, MariaYang, Ling LiLee, Matthew Meng YangYang, PeixinLee, Ming JieYang, YeongwookLee, PhilhyuYang, ZhihongLee, PosenYao, ChaoqunLee, RegentYaroslavsky, Anna N.Lee, Sung KiYayan, JosefLee, TimYeh, Hong-ZenLee, Tsung-HsienYeh, RandyLee, Wei-ChiehYeo, SujungLee, Young-kyunYerlikaya, GülenLee, Young-SilYin, ChangchuanLee, ZhenghongYin, MengLeenen, Luke L. P. H.Yip, Bon HamLei, WeiYokoi, FumiakiLeibowitz-Amit, RayaYokokawa, TetsuroLejniece, SandraYokoyama, YujiroLemstra, Afina Willemina EvelienYoneda, TakashiLenghel, Lavinia ManuelaYong, Kar WeyLeoncini, LorenzoYoo, Jae-SungLeopoulou, MariannaYoo, Jung SunLeru, PolianaYordanov, AngelLesauskaite, VaivaYoshida, HiroshiLesser, Thomas G.Yoshida, YoshioLevine, Elliot M.Yu, Jian Q.Levy, StevenYu, JinxingLezot, FredericYu, Ollie YiruLi, ChenyuYu, ShaodeLi, Chien-FengYu, YanpingLi, ChunhuiYu, ZeyunLi, DongYuan, QiLi, FuhaiYuan, ZhihongLi, KangYue, John K.Li, Mei-LingZ’Graggen, WernerLi, MengZabotti, AlenLi, RongZafar, HaroonLi, Wei-MingZafrakas, MenelaosLi, XiangZagidullin, NaufalLi, XiaopengZak, MarekLi, XinZakrocka, IzabelaLi, YingZamarchi, RitaLi, YvonneZamboni, PaoloLiang, JunZamorano-Leon, José JavierLiang, YanZamyatnin, AndreyLiao, WupengZanetti, ElisabettaLiapis, GeorgeZanin, MassimilianoLiau, Nicholas P. D.Zannoni, Gian FrancoLiberale, GabrielZaric, SvetislavLichtenauer, MichaelZauderer, Marjorie G.Lidbury, BrettZavattaro, ElisaLin, Chun-LiZavos, ChristosLin, Hsiang-YuZeleznik, Oana AlinaLin, Hsueh-chunZelger, BernhardLin, I-ChaZemouri, RyadLin, Tz-FengZenarruzabeitia, OlatzLinder, EwertZeng, XiangyanLindqvist, JohanZewdie, Ephrem TakeleLinek, PawełZhang, ChengzhiLinhartová, Petra BořilováZhang, ChiLio, DoZhang, ErshuaiLithgow, BrianZhang, HuaLitofsky, Norman ScottZhang, MingLittle, PeterZhang, QianLitvinova, LarisaZhang, XinxingLitwin, TomaszZhang, XiujuanLiu, GuanQunZhang, YanfeiLiu, GuanshuZhang, ZhaoLiu, GuigenZhang, ZhiguoLiu, Jui-MingZhao, NingningLiu, MinghuaZhao, QianLiu, NanaZhao, YueLiu, YuZheng, DandanLiu, Yueh-WeiZheng, MinzhangLiu, ZhixiaZhou, QingyuLivaniou, EvangeliaZhou, TianhaoLlobet, RafaelZhou, ZhuhuangLo Muzio, LorenzoZhurakivska, KhrystynaLobo, JoaoZibis, Aristeidis H.Lodi, AlessiaZicari, AlessandraLoellgen, HerbertZieger, MartinLoffroy, RomaricZieliński, MaciejLoghin, CatalinZiemssen, FockeLoglio, AlessandroZimran, AriLojkić, IvanaZingarelli, BasiliaLomonaco, TommasoZiółkowski, RobertLonardo, AmedeoZoli, AngeloLong, MeixiaoZolotukhin, Igor A.Longo, Dario LivioZong, WeiweiLopez-Escamez, Jose A.Zubrzycki, JarosławLópez-Mora, Diego AlfonsoZucchetta, Pietro

